# Dystrophia Smolandiensis is characterized by a novel *NQO1* variant and a distinct phenotype from *COL17A1* ‐associated epithelial recurrent erosion dystrophy

**DOI:** 10.1111/aos.70171

**Published:** 2026-06-10

**Authors:** Karl De Geer, Kristina Lagerstedt‐Robinson, Daniel Nilsson, Cilla Söderhäll, Björn Hammar, Erik Björck

**Affiliations:** ^1^ Department of Molecular Medicine and Surgery Karolinska Institutet Stockholm Sweden; ^2^ Department of Clinical Genetics and Genomics Karolinska University Hospital Stockholm Sweden; ^3^ Department of Women's and Children's Health Karolinska Institutet Stockholm Sweden; ^4^ Astrid Lindgren Children's Hospital Karolinska University Hospital Stockholm Sweden; ^5^ Department of Ophthalmology, Department of Clinical Sciences Lund Lund University, Skåne University Hospital Lund Sweden

**Keywords:** *COL17A1*, corneal dystrophy, corneal erosion, ERED, keloid, *NQO1*

## Abstract

**Purpose:**

To determine the molecular cause of the two epithelial recurrent erosion dystrophies, Dystrophia Smolandiensis and Dystrophia Helsinglandica, and to identify phenotypic differences between the two conditions.

**Methods:**

DNA samples and clinical data from structured interview records were obtained from the Swedish families in which Dystrophia Smolandiensis and Dystrophia Helsinglandica were originally characterized. Candidate variant detection was performed using combinations of linkage analysis, Sanger sequencing, exome sequencing and genome sequencing. Differences in age of onset and precipitating factors of erosive episodes were assessed using the Wilcoxon rank sum test and Fisher's exact test.

**Results:**

In Dystrophia Smolandiensis, the novel candidate variant *NQO1* c.535 T>A p.(Phe179Ile) co‐segregated with disease across 49 informative meioses. In Dystrophia Helsinglandica, the known pathogenic splice variant *COL17A1* c.3156C>T was identified. There were significant phenotypic differences between the two conditions. The median age of onset was lower in Dystrophia Smolandiensis (2.5 years vs. 6 years, *p* = 0.00065). Cigarette smoke, intense sunlight and pregnancy were distinct precipitating factors of erosive episodes in Dystrophia Smolandiensis (*p* = 0.0010, *p* < 0.0001 and *p* = 0.016, respectively), whereas minor trauma to the eye was specific to Dystrophia Helsinglandica (*p* < 0.0001).

**Conclusion:**

*NQO1* is a candidate gene for Dystrophia Smolandiensis, while Dystrophia Helsinglandica is synonymous with *COL17A1*‐associated ERED. The two conditions are characterized by distinct phenotypic differences. Based on these findings, we suggest a reclassification of epithelial recurrent erosion dystrophies guided by genetics. We propose designating *COL17A1*‐associated epithelial recurrent erosion dystrophy as ERED1 and *NQO1*‐associated epithelial recurrent erosion dystrophy as ERED2.

## 1 INTRODUCTION

Epithelial recurrent erosion dystrophies (EREDs) are a group of rare corneal dystrophies characterized by childhood onset of painful erosions of the corneal epithelium (Weiss et al., 2008). Between episodes, the cornea typically appears normal. Symptoms often diminish with age, but repeated erosions may lead to subepithelial fibrosis and visual impairment (Hammar et al., 2008, 2009; Jonsson et al., 2015; Lisch et al., 2012).

Corneal dystrophies are grouped by anatomic location according to the International Committee for the Classification of Corneal Dystrophies (IC3D). In addition, each dystrophy is assigned to one of four evidence categories based on phenotypic and molecular evidence supporting that the dystrophy is a distinct entity. Category 1 represents a well‐defined dystrophy with a known molecular cause. Categories 2 and 3 also represent well‐defined dystrophies, but in which the molecular cause is not known. Category 4 is reserved for suspected dystrophies without convincing evidence for being a distinct entity (Weiss et al., 2024).

The IC3D recognizes four EREDs: epithelial recurrent erosion dystrophy (ERED), Franceschetti corneal dystrophy, Dystrophia Smolandiensis and Dystrophia Helsinglandica. Only ERED is assigned to category 1 (Weiss et al., 2024). ERED is an autosomal dominant dystrophy caused by variants in *COL17A1* (Jonsson et al., 2015). In contrast, Franceschetti corneal dystrophy, Dystrophia Smolandiensis and Dystrophia Helsinglandica are assigned to category 4, as their molecular causes are still unknown. It has been suspected that they may represent phenotypic variants of a single *COL17A1*‐associated condition due to overlapping clinical and histopathological features with ERED (Turunen et al., 2024; Weiss et al., 2024).

However, in the original clinical characterizations of Dystrophia Smolandiensis and Dystrophia Helsinglandica, there was no hesitation during the ophthalmological examination and the descriptions of the medical history that the two conditions represented distinct phenotypes, and we therefore suggested that they likely were separate disorders. In Dystrophia Smolandiensis, the onset of erosive episodes was typically before three years of age. From age seven to midlife, half of affected individuals developed keloid‐like structures in the central cornea that reduced vision to a varying degree and sometimes required corneal grafting (Hammar et al., 2008, 2010). In contrast, the onset of Dystrophia Helsinglandica was typically between four and seven years of age. By age 37, all affected individuals developed subepithelial fibrosis in the mid‐periphery that progressed slowly toward the central cornea, reduced vision to a minor degree, and never necessitated corneal grafting (Hammar et al., 2009; Neira et al., 2010).

Thus, the question of whether Dystrophia Smolandiensis and Dystrophia Helsinglandica represent distinct diseases has remained unsolved. The aim of this study was to determine the molecular cause of Dystrophia Smolandiensis and Dystrophia Helsinglandica and to identify statistically significant phenotypic differences between the two conditions.

## 2 MATERIALS AND METHODS

### 2.1 Subjects

Before inclusion, oral and written information about the purpose of the study was provided and each participant gave their written informed consent. The principles of the Declaration of Helsinki were followed, and the study was approved by the Swedish Ethical Reviews Authority, Dnr 02–381.

Genetic and clinical data were obtained from the Swedish families in which Dystrophia Smolandiensis and Dystrophia Helsinglandica were originally characterized by Hammar et al. (Hammar et al., 2008, 2009). Peripheral blood for genetic analysis was collected from 30 affected and 15 unaffected individuals in the Dystrophia Smolandiensis family, and from 59 affected and 57 unaffected individuals in the Dystrophia Helsinglandica family. Clinical data for comparison between the two conditions were extracted from the original ophthalmological interview forms collected in 2003–2004 (*n* = 21 for the Dystrophia Smolandiensis family and *n* = 30 for the Dystrophia Helsinglandica family). The original clinical characterization of Dystrophia Helsinglandica included a total of 32 affected individuals, but two interview forms were not found in this study (Hammar et al., 2009).

### 2.2 Genetic analysis

Genomic DNA was extracted using standard protocols. In Dystrophia Smolandiensis, three individuals from different branches of the family were selected for exome analysis to identify candidate variants (Figure 1a). Libraries for exome sequencing on Illumina HiSeq2000 (Illumina, San Diego, CA, USA) were prepared from DNA samples and exome sequences were enriched with Agilent SureSelect Human All Exon 50 M (Agilent Technologies Inc., Santa Clara, CA, USA), according to the manufacturer's instructions. Post‐capture libraries were sequenced as 2×100 bp paired end reads on an Illumina sequencer. Reads were base called using offline CASAVA (v 1.7, Illumina). Sample library preparation, sequencing and initial bioinformatics up to base calling and demultiplexing were performed at the Science for Life Laboratory, Stockholm.

**FIGURE 1:**
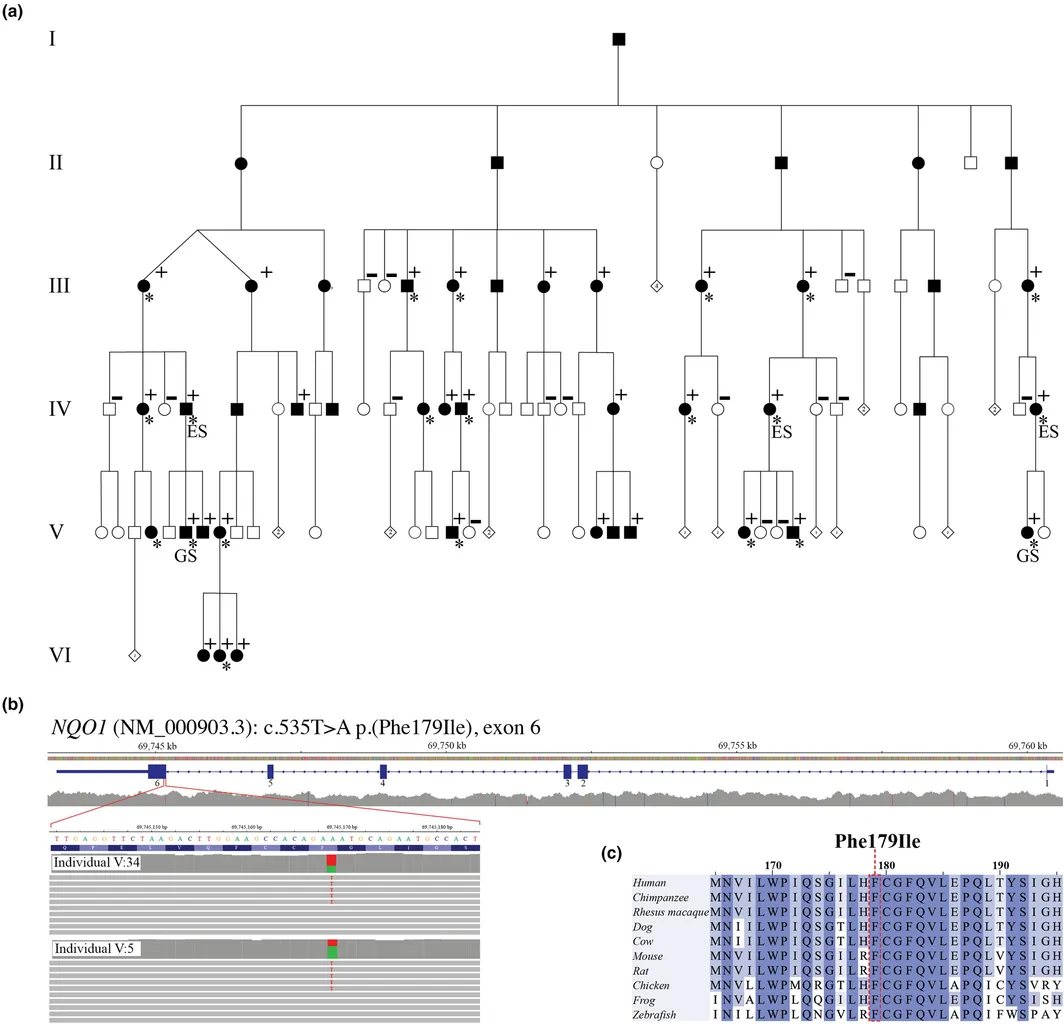
(a) Pedigree of the Dystrophia Smolandiensis family. Squares: Males; circles: Females. Filled symbols: Affected; unfilled symbols: Unaffected. Asterisk: Examined and interviewed in the original clinical characterization (Hammar et al., 2008). ES: Exome sequencing; GS: Genome sequencing. Plus sign: Heterozygous for *NQO1* p.(Phe179Ile); minus sign: Non‐carrier. (b) Representative genome sequencing reads from two affected individuals heterozygous for *NQO1* p.(Phe179Ile), visualized in Integrative Genomics Viewer. (c) Multiple sequence alignment of the corresponding region of human NQO1 with nine vertebrate orthologs.

An in‐house pipeline was used to process reads and attain candidate genes, freely available under a GPL licence (http://github.com/dnil/etiologica). Briefly, reads were aligned to GRCh37 (hg19) using Mosaik (v1.0.1388) (Lee et al., 2014). Duplicate read pairs were removed using Mosaik DupSnoop. Variants were called using the Samtools package (v.0.1.18) (Li et al., 2009). These were quality filtered (Q > 20) and annotated using ANNOVAR (version 2011 Oct 05) (Wang et al., 2010). Variants were further filtered using ANNOVAR to remove those found at a 1000 genomes minor allele frequency of 3% and above, as well as variants found in dispensable genes, truncated at a minor allele frequency of more than 1% in any 1000 genomes subpopulation, variants present in dbSNP v132 and variants not predicted damaging by PolyPhen2 (threshold of 0.7) (Adzhubei et al., 2010; Genomes Project Consortium et al., 2010). Remaining variants heterozygous in all three individuals were retained. A single candidate variant passed the filters. Segregation of the candidate variant with the disease phenotype was assessed by Sanger sequencing in both affected and unaffected family members (primers and protocols are available upon request). Sequencing products were analysed using SeqScape 2.7 (Applied Biosystems, Foster City, CA, USA).

Two additional individuals from opposite branches of the family were also selected for genome sequencing to identify a possible intronic or structural variant (SV) as a cause of Dystrophia Smolandiensis (Figure 1a). Our genome sequencing workflow has been described in detail previously (Stranneheim et al., 2021). In brief, reads were aligned to GRCh37 (hg19) and processed using the in‐house pipeline MIP (Mutation Identification Pipeline, https://github.com/Clinical‐Genomics/MIP) to detect and rank single nucleotide variants (SNVs) (including small insertions and deletions), larger SVs (insertions, deletions, inversions and translocations), and short tandem repeats (STRs). Variants were visualized using Scout, an in‐house analysis platform (Scout; https://github.com/Clinical‐Genomics/scout).

Analysis of genome sequencing data included the top 50 SNVs in all known genes as well as a separate analysis of SNVs, SVs, and STRs in a comprehensive corneal dystrophy gene panel covering 34 genes (Table S1). Variants heterozygous in both individuals, consistent with autosomal dominant inheritance, were retained. Common variants were excluded based on a minor allele frequency >1% in the gnomAD v2.1 dataset or ≥5 observations in our local database (Karczewski et al., 2020). Non‐coding variants were discarded if SpliceAI predicted no impact on splicing (Jaganathan et al., 2019). The previously identified candidate variant from the exome sequencing analysis was the only variant that passed the filters.

In the Dystrophia Helsinglandica family, a genome‐wide linkage analysis was performed to identify chromosomal regions shared among affected individuals, using 512 polymorphic microsatellite markers genotyped in 86 family members. The genotyping reactions were conducted by deCODE Genetics in Reykjavik, Iceland. The PedCheck program was used to examine the pedigree for genotyping errors and Mendelian inconsistencies (O'Connell & Weeks, 1998). All inconsistencies were re‐analysed, and incompatibilities were resolved unambiguously, or the marker was excluded. The deCODE map was used (Kong et al., 2002). Parametric multipoint LOD (logarithm of the odds) score analyses were performed using Genehunter v1.2 r3beta (Kruglyak et al., 1996). The phenotype ‘recurrent corneal dystrophy’ was set as an autosomal dominant trait with full penetrance based on the pedigree. The allele frequencies were estimated from all genotyped individuals. Allele frequencies of 0.999 for the wild‐type allele and 0.001 for the variant allele were assumed.

A candidate gene was identified in the region with the highest LOD‐score. Three affected individuals were selected for screening of the whole gene with Sanger sequencing using exon‐specific primers covering all 55 coding exons and extending at least 20 base pairs into the intronic sequences (primers and protocols are available upon request). A candidate variant heterozygous in all three individuals was identified and confirmed in three additional individuals by exome sequencing. Further co‐segregation of the variant in affected and unaffected family members was assessed by Sanger sequencing (primers and protocols are available upon request). Sequencing products were analysed using SeqScape 2.7 (Applied Biosystems, Foster City, CA, USA).

### 2.3 Protein conservation analysis

Orthologous NQO1 protein sequences from 10 vertebrate species were retrieved from UniProt: human (P15559), chimpanzee (A0A6D2WE22), rhesus macaque (A0A1D5QSF4), dog (A0A8I3NAN7), cow (A0A4W2E8H6), mouse (Q64669), rat (P05982), chicken (A0A8V0XZ95), frog (A4QNN5), and zebrafish (A0A2R8PW659). Sequences were aligned using MAFFT L‐INS‐i via Jalview (version 2.11.5.0) with the human NQO1 protein sequence as the reference for residue numbering. Conservation was visualized using the percentage identity colour scheme in Jalview.

### 2.4 Statistics

Co‐segregation analysis of the candidate variant in Dystrophia Smolandiensis was performed according to the method described by Jarvik and Browning (Jarvik & Browning, 2016). The probability that the observed segregation pattern occurred under random segregation was calculated as (1/2)^*m*^, where *m* is the number of informative meioses. A dominant model with complete penetrance, a single causal allele, and no phenocopies was assumed.

Statistical analysis of differences in age of onset and precipitating factors between Dystrophia Smolandiensis and Dystrophia Helsinglandica was possible because both families were originally characterized using identical interview forms and were examined by the same ophthalmologists and clinical geneticist (Hammar et al., 2008, 2009). Potential differences in the age at which subepithelial opacities developed could not be analysed, as the dataset recorded only the presence or absence of corneal opacities and did not include information on the timing or onset of corneal changes.

For the statistical analysis of precipitating factors of erosive episodes, only factors reported by at least 15% of individuals in either the Dystrophia Smolandiensis family or the Dystrophia Helsinglandica family were included. The prevalence of precipitating factors was calculated from the original interview forms. There were minor discrepancies compared to the prevalence reported in the original clinical characterizations, which did not significantly alter the results of the statistical analysis. The factors originally reported as ‘Dust (e.g. sawdust, metallic dust)’ in Dystrophia Smolandiensis and ‘Foreign body’ in Dystrophia Helsinglandica were combined to one factor, ‘Foreign body’. For all other precipitating factors, the names used in the original publications were maintained to facilitate interpretability between the articles (Hammar et al., 2008, 2009).

Differences in the distribution of categorical and continuous variables were assessed using Fisher's exact test and the Wilcoxon rank sum test, respectively. In a subset of patients, age of onset was reported as a timespan rather than a specific year (*n* = 20/21 in the Dystrophia Smolandiensis family and *n* = 10/30 in the Dystrophia Helsinglandica family). In those cases, the midpoint of the range was used as the age of onset. Statistical significance was defined as *p* < 0.05.

## 3 RESULTS

### 3.1 A novel *NQO1* variant segregates with disease in Dystrophia Smolandiensis

Exome sequencing of three affected individuals in the Dystrophia Smolandiensis family identified only one coding variant that passed all filters: *NQO1* NM_000903.3: c.535 T>A p.(Phe179Ile). Subsequent segregation analysis demonstrated complete co‐segregation of the variant with the disease phenotype. All 30 clinically affected individuals tested for the variant were carriers, whereas all 15 unaffected tested individuals were noncarriers. In addition, there were five affected obligate carriers (Table 1, Figure 1a). In total, there were 49 informative meioses. The probability of observing this segregation pattern under random segregation was (1/2)^49^ (≈1.78 × 10^−15^).

**TABLE 1:** Molecular characteristics of Dystrophia Smolandiensis and Dystrophia Helsinglandica.

Disease	Genetic variant	Carriers (affected)	Noncarriers (unaffected)
Dystrophia Smolandiensis	*NQO1* NM_000903.3: c.535 T>A p.(Phe179Ile)	30 (30)	15 (15)
Dystrophia Helsinglandica	*COL17A1* NM_000494.4: c.3156C>T p.(Gly1052=)	59 (59)	57 (57)

Next, genome sequencing was performed in two affected individuals from opposite branches of the family. The same *NQO1* variant was confirmed. Analysis of a comprehensive corneal dystrophy gene panel, including *COL17A1*, did not detect additional candidate variants (Figure 1b, Table S1). In silico pathogenicity prediction of the *NQO1* variant using REVEL yielded a score of 0.767 (Ioannidis et al., 2016; Pejaver et al., 2022). Potential splicing impact was assessed using SpliceAI and yielded a delta score of 0.02 (Jaganathan et al., 2019). The *NQO1* variant was absent from 807 162 samples in the gnomAD v4.1.0 dataset as of December 2025 (Karczewski et al., 2020). Protein conservation analysis showed that the Phe179 residue in human NQO1 was conserved across nine vertebrate orthologs (Figure 1c).

### 3.2 Dystrophia Helsinglandica is caused by the ERED‐associated variant *COL17A1* c.3156C>T

Linkage analysis was performed on 86 members of the Dystrophia Helsinglandica family and identified a shared locus spanning 27 Mb on chromosome 10. The locus included the markers D10S185, D10S1267, and D10S597 and was limited by D10S1753 (10q22) and D10S1693 (10q25). The LOD‐score was 8.84. This corresponded to a probability of approximately 1.4×10^−9^ that the observed segregation pattern occurred by chance.

*COL17A1* was identified as a candidate gene in the region and was selected for Sanger sequencing. Sequencing of three affected family members identified a single rare heterozygous variant, *COL17A1* NM_000494.4: c.3156C>T p.(Gly1052=). Subsequent exome sequencing of the same family members verified this finding. The variant co‐segregated completely with disease among 116 tested family members (Table 1). The variant has previously been reported as pathogenic in several independent ERED families (Jonsson et al., 2015; Lin et al., 2016; Oliver et al., 2016; Turunen et al., 2024; Vahedi et al., 2018).

### 3.3 Phenotypic differences between Dystrophia Smolandiensis and Dystrophia Helsinglandica

As shown in Table 2 and Figure 2, Dystrophia Smolandiensis had a lower median age of onset compared with Dystrophia Helsinglandica (2.5 years vs. 6 years, *p* = 0.00065, Wilcoxon rank sum test). One extreme outlier was observed in the Dystrophia Smolandiensis family, with a reported age of onset of 40 years, which markedly differed from the remaining responses.

**TABLE 2:** Statistical comparison of phenotypic features in Dystrophia Smolandiensis and Dystrophia Helsinglandica.

Category	Dystrophia Smolandiensis	Dystrophia Helsinglandica	*p*‐value
Gene	*NQO1*	*COL17A1*	
Median age of onset, years (range)	2.5 (0.5–40, *n* = 21)	6 (4–9, *n* = 30)	0.00065
Precipitating factors			
Cigarette smoke	7/21 (33%)	0/30 (0%)	0.0010
Common cold	15/21 (71%)	5/30 (17%)	0.00012
Draught (e.g. fans)	18/21 (86%)	8/30 (27%)	<0.0001
Foreign body	9/21 (43%)	6/30 (20%)	0.12
Intense sunlight	16/21 (76%)	1/30 (3%)	<0.0001
Lack of sleep	10/21 (48%)	4/30 (13%)	0.011
Minor trauma	0/21 (0%)	29/30 (97%)	<0.0001
Pregnancy	4/11 (36%)	0/17 (0%)	0.016
Upon awakening	0/21 (0%)	9/30 (30%)	0.0068
Treatment effect of Vitamin B	13/17 (76%)	N/A	

*Note*: Wilcoxon rank‐sum test was used for age of onset and Fisher's exact test for categorical variables. Pregnancy was calculated among mothers in the Dystrophia Smolandiensis family and among all females in the Dystrophia Helsinglandica family. The effect of Vitamin B‐complex was calculated among individuals that had tried the treatment. Because the treatment had not been tested in the Dystrophia Helsinglandica family, statistical comparison was not possible.

Abbreviation: N/A, not applicable.

**FIGURE 2:**
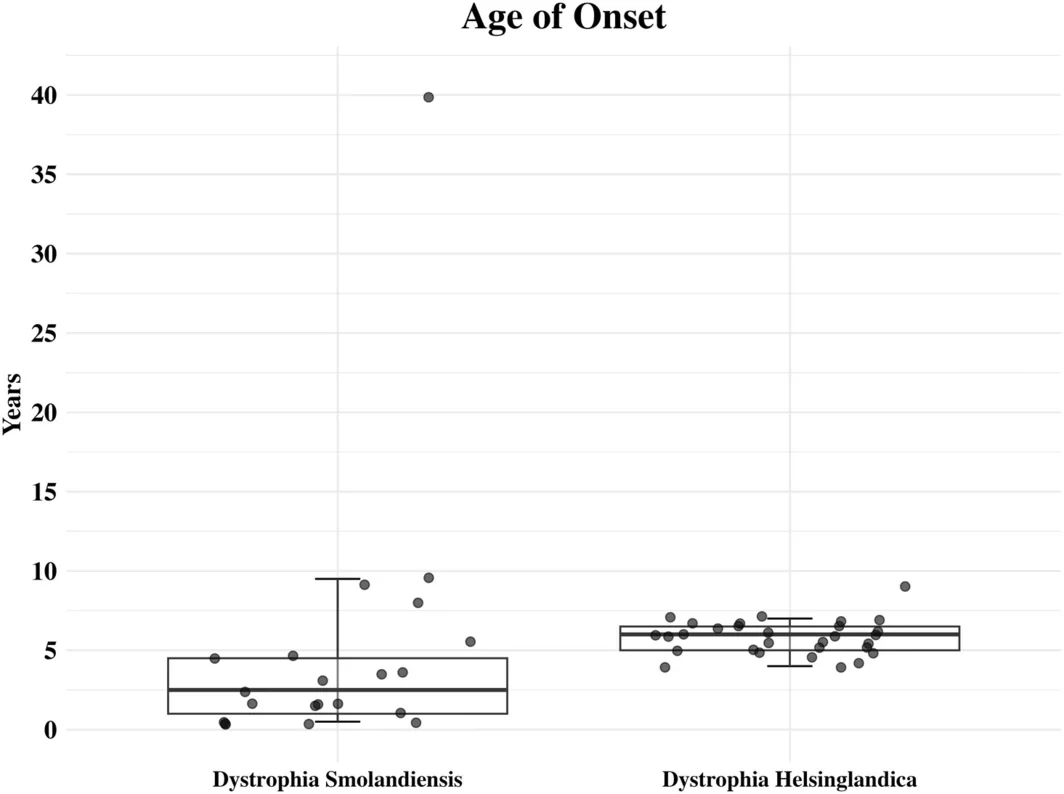
Age of onset distribution for Dystrophia Smolandiensis (*n* = 21) and Dystrophia Helsinglandica (*n* = 30). Boxes represent the interquartile range (IQR) with the median indicated by the horizontal thick lines. Whiskers extend to the most extreme data points within 1.5 x IQR of the quartiles. Circles represent individual data points. Circles beyond the whiskers indicate potential outliers.

The precipitating factors of erosive episodes in the corneal epithelium also differed between the conditions. Cigarette smoke, intense sunlight and pregnancy were common triggers in Dystrophia Smolandiensis but rare in Dystrophia Helsinglandica (*p* = 0.0010, *p* < 0.0001 and *p* = 0.016, respectively, Fisher's exact test). In contrast, minor trauma to the eye and erosive episodes upon awakening were the predominant precipitating factors in Dystrophia Helsinglandica, but were not reported by any individual in the Dystrophia Smolandiensis family (*p* < 0.0001 and *p* = 0.0068, respectively). Other precipitating factors that were more frequent in both conditions yet significantly more common in Dystrophia Smolandiensis were the common cold, draught (such as air from fans) and lack of sleep (*p* = 0.00012, *p* < 0.0001, and *p* = 0.011, respectively) (Table 2).

A positive response to treatment with vitamin B‐complex was reported by 13 of 17 (76%) that had tested the treatment in the Dystrophia Smolandiensis family, many describing minimal or no remaining symptoms. The most used preparation was B‐kombin Forte N® (ACO, Täby, Sweden), which contained thiamine, riboflavin, nicotinamide, pyridoxine, calcium pantothenate, cyanocobalamin, and folic acid (Hammar et al., 2008). A comparison with Dystrophia Helsinglandica was not possible as the treatment had not been widely tested in the family.

## 4 DISCUSSION

To date, the genetic aetiology of EREDs has been limited to pathogenic variants in *COL17A1*, which cause the category 1 corneal dystrophy ERED (Jonsson et al., 2015). However, the third edition of the IC3D additionally recognizes three EREDs classified as category 4, owing to insufficient genetic evidence: Dystrophia Smolandiensis, Dystrophia Helsinglandica, and Franceschetti corneal dystrophy (Weiss et al., 2024). In this study, we present evidence supporting *NQO1* as a candidate gene for Dystrophia Smolandiensis. We further demonstrate that Dystrophia Helsinglandica is synonymous with ERED, caused by a known pathogenic variant in *COL17A1*. We also identify statistically significant clinical differences between Dystrophia Smolandiensis and Dystrophia Helsinglandica. Collectively, these findings indicate genetic heterogeneity among EREDs. Although additional studies are required to verify the association between *NQO1* and Dystrophia Smolandiensis, our data support a reclassification of EREDs guided by genetics. We therefore propose an updated nomenclature analogous to that used for Posterior Polymorphous Corneal Dystrophies (e.g. PPCD1, PPCD3), designating *COL17A1*‐associated ERED as ERED1 and *NQO1*‐associated ERED as ERED2 (Weiss et al., 2024).

There are several reasons why we believe that the *NQO1* variant p.(Phe179Ile) is the cause of Dystrophia Smolandiensis/ERED2. First, the variant showed complete co‐segregation with disease across 49 informative meioses (Table 1, Figure 1a). If the variant was unrelated to disease, the probability of observing this segregation pattern by chance would be (1/2)^49^ (≈1.78 × 10^−15^). This represents extremely strong genetic evidence linking the variant to the Dystrophia Smolandiensis/ERED2 phenotype. Second, genome sequencing analysis did not identify alternative candidate variants as the cause of the disease and excluded known causes of corneal dystrophies (Figure 1b). Third, the variant was predicted to be pathogenic in silico by REVEL with a score of 0.767, above the commonly used cutoff of 0.75. Fourth, the variant is novel, meaning it has not been described before. It was absent from gnomAD v4.1.0, the largest database of human genetic variation, which includes more than 800 000 samples (Karczewski et al., 2020). Fifth, the phenylalanine amino acid residue at position 179 is highly conserved among vertebrates, which typically indicates that the residue is important for protein stability or function (Figure 1c). Thus, multiple lines of evidence support that the *NQO1* variant is the molecular cause of Dystrophia Smolandiensis/ERED2, although it is formally classified as a variant of unknown significance according to the criteria established by the American College of Medical Genetics and Genomics (Richards et al., 2015). The criteria PM2 (not in matched controls) and PP3 (predicted pathogenic) were fulfilled, yielding three points. The extensive co‐segregation data of the variant could not be used to apply PP1 (strong co‐segregation evidence, four points), since the criterion should not be used for the determination of novel gene‐disease associations (Biesecker et al., 2024). Identification of the variant in additional families with a similar phenotype would enable use of PP1 and reclassification of the variant as disease‐causing.

In the Dystrophia Helsinglandica/ERED1 family, we used several methods to demonstrate that the molecular cause is the known pathogenic variant *COL17A1* c.3156C>T p.(Gly1052=). First, linkage analysis identified a shared locus spanning 27 Mb on chromosome 10 that included *COL17A1*. There was no shared locus spanning *NQO1*. Second, Sanger sequencing followed by exome sequencing revealed only one rare variant within the region, *COL17A1* c.3156C>T p.(Gly1052=). Third, the variant showed complete co‐segregation with the disease among 116 family members (Table 1). Fourth, the variant has been reported previously in several ERED families with a typical age of onset between 5 and 10 years, similar to the 4–9 year range observed in Dystrophia Helsinglandica/ERED1 (Jonsson et al., 2015; Lin et al., 2016; Oliver et al., 2016; Turunen et al., 2024; Vahedi et al., 2018). Fifth, Jonsson et al. have demonstrated in vitro that c.3156C>T causes aberrant splicing through the introduction of a cryptic splice donor site, resulting in an insertion of a single amino acid, p.Gly1052Ala, followed by an in‐frame deletion of 17 amino acids, p.Gly1053_1070del (Jonsson et al., 2015). Based on these findings, we propose that Dystrophia Helsinglandica is synonymous with ERED.

Given the findings that Dystrophia Smolandiensis/ERED2 and Dystrophia Helsinglandica/ERED1 have different molecular causes, we re‐examined the clinical data from the original descriptions of the diseases (Hammar et al., 2008, 2009). Prior to the molecular findings, there was uncertainty as to whether the clinical differences truly represented two separate conditions. We could statistically show that the Dystrophia Smolandiensis/ERED2 phenotype is distinct from the phenotype of Dystrophia Helsinglandica/ERED1, characterized by a significantly younger age of onset and different precipitating factors of erosive episodes (Table 2). These findings may facilitate clinical diagnostics of EREDs and guide molecular testing. Dystrophia Helsinglandica/ERED1 should be suspected if erosive episodes are precipitated by minor trauma to the eye or triggered upon awakening. In contrast, precipitation by exposure to cigarette smoke, intense sunlight, and pregnancy should raise suspicion for Dystrophia Smolandiensis/ERED2, and treatment with vitamin B‐complex may be tested.

We do not yet understand the mechanism underlying the reported treatment effect of vitamin B‐complex in Dystrophia Smolandiensis/ERED2, but the effect is intriguing in relation to the *NQO1* variant. *NQO1* encodes the antioxidant enzyme nicotinamide adenine dinucleotide phosphate (NAD(P)H):quinone oxidoreductase 1 (NQO1), which is highly expressed in the corneal epithelium (Schelonka et al., 2000; Siegel & Ross, 2000). NQO1 detoxifies quinones via two‐electron reduction, preventing the formation of semiquinone radicals and reactive oxygen species (ROS) (Lind et al., 1982). The activity of NQO1 depends on the coenzymes flavin adenine dinucleotide (FAD) and NAD(P)H, derived from vitamin B_2_ and vitamin B_3_, both of which are present in the treatment used in Dystrophia Smolandiensis/ERED2 (Ross & Siegel, 2021). We hypothesize that the *NQO1* p.(Phe179Ile) variant alters enzymatic activity, and that this activity may be restored through coenzyme supplementation.

It is of further interest to note possible connections between the *NQO1* variant and factors reported to precipitate erosive episodes. Both cigarette smoke and sunlight exposure have been shown to induce oxidative stress and upregulate *NQO1* expression in the mouse cornea (Jin et al., 2021; Liu et al., 2020; Zhang et al., 2022). Notably, sunlight is predominantly absorbed by the central cornea, a finding of particular relevance given the central corneal opacities characteristic of Dystrophia Smolandiensis/ERED2, in contrast to the more peripheral opacities observed in Dystrophia Helsinglandica/ERED1 (Doutch et al., 2012; Hammar et al., 2010; Neira et al., 2010). Consistent with this anatomical distinction, intense sunlight was frequently reported as a precipitating factor of erosive episodes in Dystrophia Smolandiensis/ERED2 (76%), but was rarely reported in Dystrophia Helsinglandica/ERED1 (3%). This suggests a hypothetical model in which sunlight‐induced oxidative stress in the central cornea, combined with a potentially impaired NQO1 response, contributes to erosive episodes and the subsequent development of keloid‐like formations in Dystrophia Smolandiensis/ERED2. Furthermore, NQO1 appears to have an important function in the cornea during states of increased oestrogen metabolism, such as pregnancy (Tulchinsky et al., 1972). In an *Nqo1*‐null mouse model of Fuchs endothelial corneal dystrophy, disease was exacerbated in female mice and the phenotype could be recapitulated in male mice by exogenous oestrogen injection (Kumar et al., 2024). Taken together, these observations suggest that precipitating factors may induce oxidative stress and upregulate *NQO1* in the corneal epithelium, which, in the presence of the variant p.(Phe179Ile), leads to erosive episodes.

This prompts the question of how p.(Phe179Ile) may affect NQO1 function. A loss‐of‐function mechanism would limit *NQO1* expression to the wild‐type allele. This is unlikely to cause the Dystrophia Smolandiensis/ERED2 phenotype, as homozygosity for the loss‐of‐function allele Pro187Ser is relatively common in the general population and has not been associated with corneal disease (Ross & Siegel, 2021). An alternative hypothesis is a toxic gain‐of‐function mechanism, whereby p.(Phe179Ile) shifts the catalytic balance of NQO1 toward one‐electron reduction, increasing ROS production rather than preventing it. Similar effects have been reported for single amino‐acid substitutions in related flavoenzymes (Anusevicius et al., 2005). In addition, structural studies have shown that the aromatic ring of phenylalanine at position 179 interacts with the coenzymes FAD and NAD(P)H to enable efficient two‐electron transfer (Asher et al., 2006; Čėnas et al., 2021; Li et al., 1995). In Dystrophia Smolandiensis/ERED2 the aromatic ring is lost due to the substitution of phenylalanine by isoleucine, which may disrupt that interaction. Future functional studies will be required to test this hypothesis.

In conclusion, we identify *NQO1* p.(Phe179Ile) as a candidate variant for Dystrophia Smolandiensis/ERED2. To our knowledge, this is the first association of *NQO1* with monogenic disease. We also show that Dystrophia Helsinglandica/ERED1 is caused by a known pathogenic variant in *COL17A1*. Furthermore, we demonstrate significant clinical differences between the conditions, supporting their classification as distinct dystrophies. Based on these findings, we propose a reclassification of EREDs guided by genetics, designating *COL17A1*‐associated ERED as ERED1 and *NQO1*‐associated ERED as ERED2.

## CONFLICT OF INTEREST STATEMENT

The authors declare no conflicts of interest.

## Supporting information


**Table S1.** Corneal dystrophy gene panel.
